# Evaluating hearing outcome, recidivism and complications in cholesteatoma surgery using the ChOLE classification system

**DOI:** 10.1007/s00405-020-06208-z

**Published:** 2020-07-13

**Authors:** David Bächinger, Adrian Rrahmani, Nora M. Weiss, Robert Mlynski, Alexander Huber, Christof Röösli

**Affiliations:** 1 Department of Otorhinolaryngology, Head and Neck Surgery, University Hospital Zurich, Frauenklinikstrasse 24, 8091 Zurich, Switzerland; 2 University of Zurich, Zurich, Switzerland; 3 Department of Otorhinolaryngology, Head and Neck Surgery “Otto Körner”, Rostock University Medical Center, Rostock, Germany

**Keywords:** Middle ear surgery, Chronic otitis media, Cholesteatoma, Pure-tone average, Air bone gap, Staging

## Abstract

**Purpose:**

To establish a standardized reporting system of cholesteatoma, the ChOLE classification has recently been introduced. We here aimed to systematically investigate the association between the ChOLE classification and (i) hearing, (ii) recidivism rate, and (iii) postoperative complications. These data may increase the utility of the ChOLE classification in clinical practice and research by stratifying patients according to expected outcomes or risks for complications.

**Methods:**

In this prospective multicentric study, we included adult patients undergoing tympanomastoid surgery due to cholesteatoma. Main outcome measures included the association of the ChOLE classification system with (i) audiometric data including air conduction (AC) and bone conduction (BC) pure-tone average (PTA), and the air–bone gap (ABG), (ii) recidivism and complication.

**Results:**

A total of 160 patients suffering from cholesteatoma were included. ChOLE stage distribution was stage I in 23 (14%), stage II in 128 (80%), and stage III in 9 (6%) patients. The ChOLE stage was associated with the postoperative AC PTA (*p* = 0.05) and the postoperative BC PTA (*p* = 0.02). Further, the status of the ossicular chain after surgery (ChOLE subdivision “O”) was associated with both the postoperative ABG (*p* = 0.0001) and the postoperative AC PTA (*p* = 0.003). Moreover, we found an association between complications (ChOLE subdivision “L) and both the postoperative BC PTA (*p* = 0.04) and the postoperative ABG (*p* = 0.04). No association between the ChOLE stage was found to both cholesteatoma recidivism and surgical complications.

**Conclusion:**

The ChOLE classification is a new system to classify cholesteatomas. We provide evidence that hearing outcomes vary among different ChOLE stages. In particular, hearing outcomes are associated with the ChOLE subdivision “O” and “L”. Thus, the ChOLE classification system has a predictive value regarding hearing outcomes.

## Introduction

*Otitis media chronica cholesteatomatosa* (OMCC) is a chronic inflammation of the middle ear with formation of cholesteatoma. By definition, cholesteatoma is an epidermoid spreading of keratinizing squamous epithelium of the outer tympanic membrane into the middle ear cavity [1]. This process results in a chronic inflammation leading to destruction of middle ear structures and the surrounding bone. Cholesteatomas are divided into congenital and aquired cholesteatoma [2]. Congenital cholesteatoma is a congenital displacement of squamous epithelium during the embryonal phase, while acquired cholesteatoma describes invasion of keratinizing squamous epithelium from the external auditory canal or the tympanic membrane into the middle ear after birth. Despite being a common otologic diagnosis, there are still large differences in the definition, classifications and management of cholesteatomas [3]. Yet, standardized reporting of cholesteatoma characteristics and surgical outcomes is a prerequisite for comparison of surgical techniques and outcomes among centers in an international setting.

To establish a standardized reporting system of cholesteatoma, there have been several attempts to categorize and classify cholesteatoma using general classifications as developed by the European Academy of Otology and Neurotology (EAONO) or the Japanese Otological Society (JOS). These two societies eventually published the “EAONO/JOS Joint Consensus Statements on the Definitions, Classification a Staging of Middle Ear Cholesteatoma” in 2016 [4]. Yet, the EAONO/JOS joint consensus has several shortcomings, as previously pointed out [5]. The EAONO/JOS joint consensus classification is primarily based on cholesteatoma extension and complications. However, apical or supra/infralabyrinthine cholesteatomas cannot be classified, and the status of the ossicular chain as well as the degree of mastoid pneumatization and ventilation is not included [4]. In an attempt to overcome these drawbacks, the ChOLE classification has been developed [5]. The ChOLE classification is based on cholesteatoma extension, ossicular chain status (as partially adopted form classification by Kartush [6] and Fisch [2]), complications as well as degree of mastoid pneumatization and ventilation. A numeric rule is used for staging, whereby stages range from stage I to stage III.

In a small cohort (*n* = 24), preliminary evidence has been found that hearing outcomes in cholesteatoma surgery are associated with the ChOLE classification [5]. In particular, the ossicular chain status (O) tended to correlate with the pre- and postoperative air–bone gap (ABG). However, no study has systematically investigated the association of hearing and the ChOLE classification up to date. Further, there are no data on the relationship of the ChOLE classification and both recidivism rate and postoperative complications. These data may increase the utility of the ChOLE classification in clinical practice and research by stratifying patients according to expected outcomes or risks for complications. Therefore, in the present study, we aimed to investigate the relationship between the ChOLE classification system and hearing outcomes, recidivism rates as well as complication rates.

## Methods

### Compliance with ethical standards

The study protocol was approved by the local Ethics Committees in accordance with the Helsinki declaration and its amendments (No. 2016-02216, Kantonale Ethikkommission, Zurich, Switzerland; No. A2017-0101, Rostock, Germany). Informed consent was obtained from all the participants.

### Patients

Patients were consecutively recruited from two tertiary referral centers between January 2016 and November 2018. Inclusion criteria were a clinical diagnosis of cholesteatoma and a minimum age of 18 years. Patients underwent extirpation of the cholesteatoma via a retroauricular access including a mastoidectomy and tympanoplasty. Autologous reconstruction material (local pediculed muscle flaps, bone paté, temporal muscle fascia and cartilage) was used to obliterate the open mastoid cavity and/or to reconstruct the posterior canal wall. In some patients, ossicular chain reconstruction (OCR, i.e., ossiculoplasty [2]) was performed during primary surgery. If the malleus (with or without head) and stapes were present, OCR was performed using autologous or titanium incus interposition. In the other cases, a titanium partial or total ossicular replacement prosthesis was used for OCR, depending on the individual ossicular defect.

### Clinical data collection

From the clinical record, demographic data were extracted. Complications refer to complications occurring up to one year after surgery. Complications include new tinnitus or vertigo, facial paralysis, liquorrhoe, infection, stenosis of the external auditory canal, perforation of the tympanic membrane, and severe postoperative otalgia. Recidivism was defined as clinically diagnosed reoccurrence of cholesteatoma at the same or a different location within one year [7].

### Pure-tone audiometry

Pure-tone audiometry was performed following standard procedures in accordance with ISO 8253-1 during the routine preoperative visit and at the postoperative visit 6–12 months after surgery. Pure-tone average (PTA) was calculated from the hearing threshold levels at 0.5, 1, 2 and 4 kHz. The ABG refers to the difference between air conduction (AC) PTA and bone conduction (BC) PTA. The BC PTA was set to 120 dB if the patient did not detect any tone in all the tested frequencies. The difference between the pre- and postoperative measurement is referred to as “shift”.

### ChOLE classification

Cholesteatomas were classified and staged according to the recently introduced ChOLE classification [5]. Briefly, cholesteatomas are classified by (i) extension with subdivisions Ch1 describing very limited extension within the middle ear to Ch4 describing a petrous apex cholesteatoma and (ii) the status of the ossicular chain with O0 indicating an intact ossicular chain to O4 indicating an ossicular defect with fixed stapes only. Of note, the O subdivision describes the status of the ossicular chain after extirpation of the cholesteatoma, but before OCR. Cholesteatomas are further classified by (iii) complications with L2 describing extracranial and L4 describing intracranial complications, and (iv) Eustachian tube functions as determined by the degree of mastoid pneumatization and ventilation with E0 indicating a good to E2 indicating a poor pneumatization and ventilation. After determining the ChOLE subdivisions, staging into three different stages (I–III) is performed using a numeric rule. The ChOLE classification was determined based on intraoperative findings (subdivisions Ch, O and L) and preoperative CT imaging (subdivision E) using a freely available online software tool [8]. Of note, subdivision L refers to complications of the cholesteatoma itself as assessed intraoperatively, while specific complications mentioned in the subsection “Clinical data collection” above refer to postoperative complications occurring within one year after surgery.

### Statistical analysis

All statistical tests were selected before data collection. Cholesteatoma recidivism was compared to ChOLE stage, cholesteatoma extension (Ch) and mastoid pneumatization/ventilation (E). Further, ChOLE stage, cholesteatoma extension (Ch), the status of the ossicular chain (O) and complications (L) were compared with each the postoperative AC PTA, postoperative BC PTA and postoperative ABG. The ChOLE stage was compared to the AC PTA shift and BC PTA shift. Complications were compared to the ChOLE stage and each of the four ChOLE subdivisions.

Statistical analyses were performed using RStudio (version 1.2.1335) [9]. The significance level was set to *p* < 0.05. To analyze whether cholesteatoma recidivism is more common in larger cholesteatomas or poor pneumatization/ventilation of the mastoid, Fisher’s test was used. A Kruskal–Wallis test was used to assess the relationship between the ChOLE classification and the hearing outcomes. If a statistically significant difference among the groups was found, post hoc testing including Dunn’s test to correct for multiple comparison was carried out. If not otherwise specified, values are reported as absolute number and percentage, mean with SD or median with the 10th and 90th percentile.

## Results

A total number of 166 patients were recruited, of which six patients were excluded because of missing values for the subdivision of the ChOLE classification or missing follow-up. Therefore, data from 160 patients were included in the final analysis. From 160 patients with cholesteatoma, 69 (43%) were female and 91 were male (57%). The mean age was 38.7 years (SD 18.8). In 87 patients (54%), the left ear was affected, in the remaining 73 patients (46%), the right ear was affected. OCR at the primary surgery was performed in 22 (14%) patients. Cholesteatoma recidivism was diagnosed in 16 patients (10%). Regarding complications, new tinnitus and new vertigo each were observed in 19 (12%) patients. A transient facial paralysis and postoperative leak of cerebrospinal fluid each were observed in 1 (0.6%) patient. Surgical site infections occurred in 23 (14%) patients and severe postoperative otalgia was found in 9 (6%) patients. Postoperatively, a (re-)perforation of the tympanic membrane occurred in 5 (3%) patients and external auditory canal stenosis developed in 15 (9%) patients.

### Audiometric outcomes

Due to missing audiometric data in five patients, analyses involving audiometric data were carried out in 155 patients. Audiometry was performed at a mean follow-up time of 9.7 (SD 2.9) months. The median preoperative AC PTA was 45.0 dB HL (10th–90th percentile: 23.8–68.8 dB HL). The median preoperative BC PTA was 15.0 dB HL (10th–90th percentile: 2.5–39.5 dB HL). The median preoperative ABG was 26.3 dB (10th–90th percentile: 11.3–43.8 dB). Postoperatively, the median AC PTA and BC PTA were 46.3 dB HL (10th–90th percentile: 26.3–80.0 dB HL), and 15.0 dB HL (10th–90th percentile: 3.8–43.8 dB HL), respectively. The median ABG for the postoperative hearing was 30.0 dB (10th–90th percentile: 13.8–47.0 dB). One patient became deaf after surgery. In this case, a ChOLE stage III cholesteatoma (Ch4a O1 L2 E2) with infra- and intralabyrinthine extension was diagnosed.

### ChOLE classification

ChOLE stage I cholesteatoma was found in 23 (14%), ChOLE stage II cholesteatoma was found in 128 (80%), and ChOLE stage III cholesteatoma was found in 9 (6%) patients. Regarding cholesteatoma extension (Ch), Ch1 was found in 42 (26%), Ch2 in 71 (44%), Ch3 in 37 (23%) and Ch4 in 10 (7%) of the patients. Assessing the status of the ossicular chain at the end of surgery (O), O0 was found in 17 (11%), O1 in 97 (61%), O2 in 36 (23%), O3 in 7 (3%) and O4 in 3 (2%) of the patients. Classifying complications (L), L0 was found in 149 (93%), L2 in 11 (7%) and L4 in none of the patients (note that there is no L1 and no L3 subdivision). Assessing eustachian tube function as determined by the degree of mastoid pneumatization and ventilation (E), E0 was found in 24 (15%), E1 in in 75 (47%), E2 in 61 (38%) of the patients.

### Association of cholesteatoma recidivism and the ChOLE classification

**Fig. 1:**
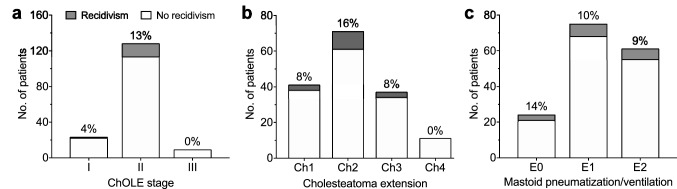
**a–c** Relationship of the ChOLE classification and cholesteatoma recurrence. Cholesteatoma recurrence was compared to the ChOLE stage (**a**), extension of cholesteatoma (ChOLE subdivision Ch; **b**), and the degree of pneumatization and ventilation of the mastoid bone (ChOLE subdivision E; **c**). Percentages refer to percentage of patients experiencing cholesteatoma recurrence

To analyze whether cholesteatoma recidivism is more common in larger cholesteatomas and poor pneumatization/ventilation of the mastoid, recidivism rates were compared to the ChOLE stage and the divisions Ch and E. No association was found between the recidivism rate and (i) the ChOLE stage (*p* = 0.33, Fig. 1a), (ii) the cholesteatoma extension (Ch) (*p* = 0.39, Fig. 1b), and (iii) the degree of pneumatization and ventilation of the mastoid bone (*p* = 0.9, Fig. 1c).

### Association of the postoperative hearing and the ChOLE classification

**Fig. 2:**
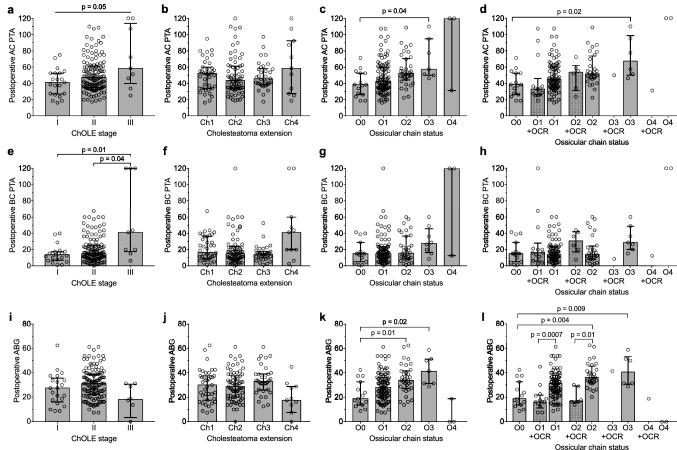
Association of audiometric outcomes and the ChOLE stage as well as the ChOLE subdivisions Ch and O. **a–d** Postoperative air-conduction pure-tone average compared to the ChOLE stage (**a**) and the ChOLE subdivisions Ch (**b**) and O (**c**, **d**). Regarding subdivision O, a post-hoc analysis was performed grouping patients according to whether primary ossicular chain reconstruction (OCR) was performed (“ + OCR”) or not (**d**). This applies also to subfigure **h** and **l**. **e–h** Postoperative bone-conduction pure-tone average compared to the ChOLE stage (**e**) and the ChOLE subdivisions Ch (**f**) and O (**g**, **h**). **i–l** Postoperative air–bone gap compared to the ChOLE stage (**i**) and the ChOLE subdivisions Ch (**j**) and O (**k**, **l**). Note that two deaf patients are included in all subfigures, which are represented by an air- and bone-conduction of 120 dB resulting in an ABG of 0 dB. Bars represent median, error bars indicate interquartile range. Groups with n < 5 were excluded from statistical analyses. In subfigures 2**d**, 2** h** and 2** l**, comparisons were only performed among non-reconstructed (O0, O1, O2, O3, O4) and among reconstructed (O1 + OCR, O2 + OCR, O3 + OCR, O4 + OCR) ears

The postoperative AC PTA showed a trend towards increasing thresholds with increasing ChOLE stage (*p* = 0.05, Fig. 2a). No association was found between the postoperative AC PTA and the extension of the cholesteatoma (Ch) (*p* = 0.63, Fig. 2b). In contrast, the status of the ossicular chain after the surgery (O) was strongly associated with the postoperative AC PTA (*p* = 0.003, Fig. 2c). In particular, we observed a trend of an increased postoperative AC PTA with increasing O status, while post hoc testing revealed a statistically significant difference between status O0 and O3 (*p* = 0.04). This effect could still be observed in a post hoc analysis grouping patients according to whether primary OCR was performed or not (Fig. 2d). Yet, within the individual O subdivisions, this post hoc analysis revealed no differences between patients who underwent primary OCR and those who did not.

The postoperative BC PTA was associated with the ChOLE stage (*p* = 0.02). In particular, post hoc testing revealed that the postoperative BC PTA is significantly different between ChOLE stage I vs. III (*p* = 0.01) and II vs. III (*p* = 0.04, Fig. 2e). The postoperative BC PTA was neither associated with the cholesteatoma extension (Ch) (*p* = 0.06, Fig. 2f) nor the ossicular chain status (O) (*p* = 0.17, Fig. 2g, h). Further, we found an association between the ChOLE subdivision covering complications (L) and the postoperative BC PTA. Patients classified as L0 had a lower median postoperative BC PTA than patients classified as L2 (median difference 14.4 dB, *p* = 0.04).

The postoperative ABG was overall significantly different among the ChOLE stages (*p* = 0.04) with post hoc comparison revealing no significant differences between any two groups (Fig. 2i). The postoperative ABG was not associated with cholesteatoma extension (Ch) (*p *= 0.06, Fig. 2j), but strongly associated with the status of the ossicular chain after surgery (O) (*p* = 0.0001, Fig. 2k). For the latter comparison, significant differences in the postoperative ABG were revealed between ossicular chain status O0 vs. O2 (*p* = 0.01) and O0 vs. O3 (*p* = 0.02). When grouping patients according to whether primary OCR was performed or not, the latter effect could still be observed among patients that did not undergo primary OCR (Fig. 2l). However, within subdivisions O1 and O2, patients who underwent primary OCR had a significantly smaller median ABG than patients who did not (*p* = 0.0007 within O1, *p* = 0.01 within O2).

Lastly, we found an association between the ChOLE subdivision covering complications (L) and the postoperative ABG. Patients classified as L0 had a smaller median postoperative ABG than patients classified as L2 (median difference 10.0 dB, *p* = 0.04).

### Association of hearing changes and ChOLE stage

**Fig. 3:**
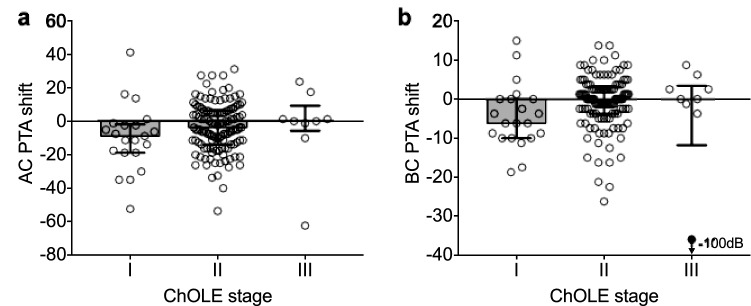
**a–b** Shift in air-conduction (**a**) and bone-conduction (**b**) in association to the ChOLE stage. Bars represent median, error bars indicate interquartile range. In (**b**), the black dot indicates audiometric data of one patient with a cholesteatoma exhibiting intralabyrinthine extension who became deaf after surgery

There was no association between the ChOLE stage and the difference of pre- and postoperative hearing (AC PTA shift; *p* = 0.14, Fig. 3a). In contrast, the ChOLE stage showed a significant association with the difference of the pre- and postoperative BC PTA (BC PTA shift; *p* = 0.03, Fig. 3b).

### Association of the sum of complications and the ChOLE classification

A higher ChOLE stage was not associated with a higher number of complications (*p* = 0.42) or a higher value in the ChOLE subdivision Ch (*p* = 0.66), O (*p* = 0.86), L (*p* = 0.13) or E (*p* = 0.93).

## Discussion

This is the first study investigating hearing outcomes and complication rates of surgical treatment of cholesteatoma based on the ChOLE classification, a standardized staging and classification system for cholesteatoma surgery. The most significant results were the association of the ChOLE stage with both the postoperative AC and BC PTA. Further, the ossicular chain after surgery (ChOLE subdivision O) was strongly associated with both the postoperative ABG and the postoperative AC PTA.

Several studies over the last decades reported factors affecting hearing outcomes of tympanomastoid surgery for cholesteatoma [10–13]. In conclusion, most prominently, a preserved stapes suprastructure predicts a favorable hearing outcome. In contrast, large perforations, otorrhea and an absent malleus handle predict an unfavorable hearing outcome. These findings highlight the importance of the ossicular chain regarding the postoperative hearing outcome. In accordance, we found that the ChOLE subdivision O describing the ossicular status after surgery was strongly associated with both the postoperative ABG and the postoperative AC PTA. In a preliminary analysis on 24 patients, Linder et al. reported a trend towards a larger ABG with increasing O subdivision of the ChOLE score [5]. We could confirm this finding in our cohort and show statistically significant differences between O0 vs. O2 as well as O0 vs. O3. Also, Linder et al. reported an ABG closure < 20 dB in all of the patients exhibiting a O0 status while this rate was lower in patients exhibiting O1, O2 or O3 [5]. In accordance, we found a median postoperative ABG of less than 20 dB in patients exhibiting an intact ossicular chain (subdivision O0), while an ossicular defect classified as O1-O4 was associated with a postoperative median ABG around 40 dB, if no OCR was performed. However, primary OCR in ossicular chain defects classified as O1 and O2 resulted in a median postoperative ABG below 20 dB, which is comparable to the postoperative ABG in patients with an intact ossicular chain.

Recently, prognostic factors for short-term hearing outcomes of ossicular chain reconstruction for “pars flaccida cholesteatoma” as classified by the EAONO/JOS staging system have been reported [14]. The rate of successful hearing improvement significantly decreased with increase in EAONO/JOS stage. Hearing outcomes were favorable if the stapes was not involved in the disease process. This corresponds well to our finding of significantly different postoperative ABG in ossicular chain statuses O0 (intact ossicular chain) vs. O2 (malleus and footplate only) as well as O0 vs. O3 (mobile footplate only).

No association was found between the recidivism rate and (i) the ChOLE stage, (ii) the degree of cholesteatoma extension or (iii) a lower degree of pneumatization. We are not aware of any studies systematically investigating the effect of cholesteatoma extension and the degree of mastoid pneumatization on residual cholesteatoma or cholesteatoma recidivism. Our findings may simply indicate that cholesteatomas, regardless of the ChOLE stage, underwent adequate surgical treatment. This may be further corroborated by the observation that no association between either the ChOLE stage or the ChOLE subdivisions was found to both cholesteatoma recidivism and complications. However, we hypothesize that these analyses may be biased by the fact that cholesteatoma extension and the degree of mastoid pneumatization itself guide the type of surgical approach and technique [2], which may act as a confounder. Moreover, since it was not the aim of this study to compare surgical techniques, the study lacks the statistical power for such a comparison. This issue should be addressed in a larger patient cohort with a longer follow-up.

Our study has several limitations. First, we observed a relatively high proportion of ChOLE stage II cholesteatomas. We speculate that this is due to the low barrier access to health care in our developed countries, where extensive cholesteatomas and cholesteatoma complications show a decreasing incidence in contrast to developing countries [15, 16]. Second, only a small proportion of supra-/infralabyrinthine and petrous apex cholesteatomas (ChOLE subdivision Ch4) was observed in our cohort, which hampers the power of the statistical analyses concerning Ch4. Yet, the observed distribution may reflect that supra-/infralabyrinthine and petrous apex cholesteatomas are a fairly small proportion of all cholesteatomas [17, 18]. Lastly, the follow-up period of one year may only cover a part of cholesteatoma recidivism [19]. Therefore, the finding of no association between cholesteatoma recidivism and neither the ChOLE stage nor the ChOLE subdivisions should be interpreted with caution.

In conclusion, the ChOLE classification is a new system to categorize cholesteatoma. This classification aims at generating comparable data sets regarding surgical outcomes. In this study, we provide evidence that hearing outcomes vary among different ChOLE stages. In particular, hearing outcomes are both associated with the ChOLE subdivision “O” and “L”. Thus, the ChOLE classification system has a predictive value regarding hearing outcomes. Future work will also investigate the association of the ChOLE classification to subjective outcomes [20]. We further encourage using the ChOLE classification with the aim of standardizing outcome research in cholesteatoma surgery by generating comparable data sets.
